# Photodynamic Therapy: A Brief History

**DOI:** 10.3390/jcm8101581

**Published:** 2019-10-02

**Authors:** David Kessel

**Affiliations:** Department of Pharmacology, Wayne State University School of Medicine, Detroit, MI 48201, USA; dhkessel@med.wayne.edu

**Keywords:** dougherty, photodynamic therapy, hematoporphyrin derivative, photofrin

## Abstract

Photodynamic therapy (PDT) involves the selective sensitization of tissues to light. A major advance in the field occurred when Thomas Dougherty at the Roswell Park Cancer Institute initiated a series of clinical studies that eventually led to FDA approval of the procedure. This report contains a summary of Dougherty’s contributions and an assessment of where this has led, along with a summary of implications for future drug development.

## 1. Introduction

The first literature reports of a ‘photodynamic effect’ were provided by Raab and von Tappeiner [1,2]. It was shown that certain dyes could sensitize microorganisms to light such that exposure to sunlight rapidly resulted in cell death. In 1948, Figge summarized a series of studies showing that exogenously-provided porphyrins selectively accumulated in murine tumors [3]. This study was later extended to include cancer patients: injection of a crude preparation of hematoporphyrin led to selective tumor fluorescence [4]. The field of clinical PDT was further advanced when a group of physicians at the Mayo Clinic reported that tumor fluorescence in patients was enhanced when a ‘derivative’ of hematoporphyrin was employed [5,6]. The nature of this material was not revealed in the initial reports. The abbreviation ‘HPD’ was used thereafter to refer to this uncharacterized hematoporphyrin derivative. Later studies revealed that it consisted of a mixture of porphyrin monomers, dimers, and higher oligomers.

## 2. The First Photosensitizers

A group at the Medical College of South Carolina reproduced the formulation of HPD and published a recipe along with a report that this agent could be used to localize neoplasia by the resulting tumor fluorescence in a large patient population [7]. The first report suggesting that this procedure might have therapeutic effects was provided by Diamond et al. [8], using a crude preparation of hematoporphyrin, with irradiation provided by fluorescent lamps (cell culture) and a 150-watt incandescent lamp, coupled to a Lucite rod, for in vivo studies. While representing somewhat crude methodology, this work did demonstrate that it was feasible to thereby eradicate glioma cells both in cell culture and transplanted tumors in mice. Following up on this study, Dougherty’s group at the Roswell Park Cancer Institute also demonstrated the ability of hematoporphyrin to eradicate murine tumors [9]. Light was provided by a 1000-watt xenon lamp, filtered to remove wavelengths below 620 and above 640 nm and focused with the aid of two convex lenses. By 1978, Dougherty’s group had turned to HPD as their photosensitizing material and reported on a large series of successful treatments of patients with tumors at various loci [10]. A report showing successful treatment of recurrent breast carcinoma followed [11]. Based on the terminology initially introduced by von Tappeiner, this procedure was termed Photodynamic Therapy (PDT). 

While the determinants of porphyrin localization in neoplastic tissues remained unknown, Dougherty and Gomer used radioactive preparations to delineate biodistribution [12]. This revealed that HPD did concentrate in malignant tissues, as compared with normal host skin and muscle, but the major sites of accumulation were liver, kidney and spleen. Since these sites were protected from irradiation, preferential eradication of malignant tissues was possible. Another important discovery identified singlet molecular oxygen, produced by the effect of incident light on porphyrins as the major cytotoxic agent produced during PDT [13]. Dougherty’s group continued to provide information on the ability of HPD for cancer control, an effort that led to approval of the procedure by the FDA for treatment of esophageal cancer in 1995. Dougherty has written a summary of the adventures that led to this critical result and other adventures before and after this event [14] and provided another biographical note in an earlier publication [15]. Critical contributors to the field included Hayata at the Tokyo Medical College, Forbes and Kaye in Australia, Carruth in England and many others in European and Asiatic countries. 

Once the first FDA approval was granted, obtaining funds for PDT research became more likely. Dougherty often recalled that his first National Institutes of Health (NIH) proposal was disapproved with one comment noting that ‘light does not penetrate living tissues’. Until 1991, there was no plausible mechanism to define the ability of photodamage to eradicate malignant cells. It was, however, appreciated that irradiation could lead to eradication of the tumor vasculature, an important element in cancer control [16]. This had an important consequence: the use of PDT to treat macular degeneration [17]. This disease results from the proliferation of extraneous blood vessels in the retina. Current protocols involve both PDT and VEGF (vascular endothelial growth factor) antagonists [18]. In the early period when it appeared that PDT alone could be a useful procedure, this led to a brief period of PDT profitability but led to the diversion of resources from studies involving cancer control. 

Until 1991, the literature relating PDT was mainly concerned with clinical reports: discussions of protocols and light sources. There was, however, a report from Pandey’s group at the Roswell Park Cancer Institute regarding the identification of the ‘active’ components in HPD [19]. These were identified as a series of porphyrin dimers and higher oligomers joined by ether linkages. The commercially-available product that had received FDA approval consisted of HPD with the monomeric porphyrins removed. This was named ‘Photofrin’ and continues to be useful for clinical PDT.

## 3. Mechanisms of Photokilling

Once it was realized that there were many agents capable of photosensitizing cells to light, it became apparent that different sub-cellular loci were being targeted depending on the photosensitizer chosen. Since most such agents are fluorogenic, the nature of this targeting was readily identified by fluorescence microscopy. It should be pointed out that the ability to photosensitize does not automatically make an agent a good candidate for PDT. With regard to drug delivery, formulation and pharmacokinetic properties are relevant with selectivity for neoplasia also an important factor. As the clinical efficacy of PDT began to be appreciated, other investigators began a search for better photosensitizing agents and for pathways to cell death in addition to vascular shutdown.

One pathway to direct photokilling was identified by Oleinick’s group at Case Western Reserve University in 1991: apoptosis [20]. This is an irreversible route to cell death that can be initiated by release of cytochrome c from photodamaged mitochondria, or from photodamage to other cellular loci. It was later observed that Bcl-2, an important protein in the apoptosis scheme was often a target for photodamage [21,22]. The 1991 report represents the first indication of a molecular mechanism that, along with vascular shut-down, could account for PDT efficacy and represents a ‘landmark’ in the field. A more circuitous route is involved when lysosomes are the PDT target. This involves release of proteases that can cleave the cytosolic protein Bid to a pro-apoptotic fragment [23], but the cell death pathway also involves apoptosis. 

A further factor in photokilling is a process termed autophagy that can either offer protection from lethal effects or, if up-regulated, may be associated with cell death. Autophagy is responsible for the ‘shoulder’ on the dose-response curve that is commonly observed [24,25], i.e., the lag in photokilling by low light doses. While necrosis can also occur, it is not clear that this is a pathway to be sought since the high PDT doses required can be non-selective. There has been a recent review on the topic of PDT-induced death pathways [26].

## 4. New Photosensitizers and Combinations

Chemotherapy is commonly delivered in the form of drug combinations with an intent to produce the maximum level of tumor eradication while not poisoning any one host system. In the context of PDT, the use of combinations of photosensitizers awaited the discovery of additional photosensitizing agents with different sub-cellular targets. While hundreds of such agents have been described in the literature, few have achieved the regulatory system approval that would be required for clinical use. When a group at the University of British Columbia synthesized a photosensitizer with better photodynamic properties than HPD, it was decided to call this ‘benzoporphyrin derivative’ (BPD) [27]. The success of this agent in ophthalmic medicine turned out to be one of the major advances in the realm of PDT, as is described below. A second advance occurred when Malik reported that administration of 5-aminolevulinic acid (ALA) could promote enhanced synthesis of protoporphyrin IX which, like HPD, had an affinity for neoplastic loci [28]. Kennedy’s group in Canada was among the first to appreciate the potential for use of ALA in clinical PDT [29]. Another agent currently receiving clinical attention in Asia is the pheophorbide HPPH that was synthesized by Pandey’s group at the Roswell Park Cancer Institute [30]. Sales of topical agents now exceed the income derived from Photofrin, demonstrating the success of dermatologic applications.

Reports on the use of photosensitizers in combination are scarce. One remarkable report from 1996 showed that a combination of BPD and the thiazine termed EtNBS could eradicate 1 cm thick tumors in the rat [31]. This was perhaps not sufficiently appreciated at the time, but Brian Wilson had calculated that the maximum depth of significant light penetration into typical tumor tissues was at best 3–5 mm [32]. It was initially thought that the efficacy of this combination could be traced to simultaneous effects on both tumor and tumor vasculature. The explanation was provided 20 years later when it was realized that these agents targeted mitochondria and ER (BPD) and lysosomes (EtNBS). Lysosomal photodamage could release calcium which, in turn, activated the enzyme calpain that cleaved the autophagy-associated protein ATG5 into a pro-apoptotic fragment [33]. As a result, this combination significantly enhanced the ability of a given light dose to produce an apoptotic response [34]. Since BPD and EtNBS are activated at different wavelengths (690 and 660 nm, respectively), use of this combination requires two light sources, a potential disadvantage. Fortunately, a formulation modification can add lysosomes to the targeting profile of BPD, as described below. Other groups have established the ability of PDT to enhance the efficacy of chemotherapy and ionizing radiation [35,36,37]. There have been recent advances relating to the ability of PDT to promote immunologic responses [38,39,40]. 

## 5. Formulations and Optimization

The original photosensitizers, HPD and Photofrin, are readily soluble in water and require no special formulation. BPD is, however, insoluble in water and must be delivered in liposomal form [41]. This preparation targets mitochondria and ER, but the targeting profile can be changed so as to reach only lysosomes by covalently binding BPD to a lipid before encapsulation in liposomes [42]. This provides a means for targeting ER, mitochondria and lysosomes with a single agent requiring activation by a single wavelength of light. Use of nanoformulations may provide a better means for both formulating photosensitizers and providing a better targeting of neoplastic lesions [43,44,45]. 

It was fortuitous that the first photosensitizer to achieve clinical use (HPD, Photofrin) was both water-soluble, targeted both tumor and tumor vasculature, was relative non-toxic and readily formulated. There were, however, significant drawbacks. There was a persistent photosensitization of skin requiring keeping treated patients away from bright lights for several weeks [11]. Moreover, the absorbance band farthest in the red, to which tissues are preferentially transparent [32] occurs at 630 nm and is relatively weak. Newer products, e.g., BPD, show much greater absorbance farther into the ‘transparency’ spectrum: 690 nm. But HPD was sufficiently active to eventually receive FDA approval for certain indications. There is considerable discussion about the properties of the ‘ideal’ photosensitizer. This agent would need to be readily formulated, stable to storage, preferentially partition into neoplastic lesions and show a significant absorbance band at the longer wavelengths. It is only lately that sub-cellular targeting has been shown to be another important factor. This could have major implications for optimizing PDT efficacy.

The ability of simultaneously targeting mitochondria and lysosomes for photodamage was discussed above. This can markedly promote the ability of light to evoke lethal photodamage. What is now being appreciated is that targeting the endoplasmic reticulum (ER) can bring about a new mode of cell death (paraptosis) that can affect cells with an impaired apoptotic program. This is evoked after ER photodamage and results in formation of an extensive series of cytoplasmic vacuoles that leads to cell death [43]. Since the targeting profile of Photofrin, HPPH and BPD includes the ER [43], this effect may account for the broad efficacy of PDT for cancer control.

## 6. Implications

This brief review has included only a fraction of the thousands of reports relating to PDT that have been published. PDT has entered the era of nanotechnology [44,45]. Several reviews, along with a summary of the early literature, have also been published [46,47,48,49,50]. The likelihood that support and continued interest in this work would have been maintained without the initial efforts of Dougherty to bring PDT to the attention of the FDA, the NIH and workers world-wide (but especially in Japan) seems remote. The most recent International Photodynamic Association conference brought almost 500 people to Boston to discuss current research prospects. Reports published in this journal and elsewhere provide some indication of the diversity of topics. Progress in therapeutic PDT is now occurring worldwide including South American, Asian and European clinics. While Dougherty was not directly involved in assessing mechanisms of photokilling or design of new photosensitizing agents, he was critical to the development of photodynamic therapy as a topic for both pre-clinical and clinical research. While many studies prior to the mid-1970s provided the basis for what was to come, his efforts made the difference between PDT being a ‘laboratory curiosity’ and significant addition to the biomedical field.
